# A Text-Book on Modern Materia Medica and Therapeutics

**Published:** 1906-02

**Authors:** 


A TEXT-BOOK ON MODERN MATERIA MEDICA AND THERAPEUTICS. By A. A. Stevens A. M., M.D., Lecturer on Physical Diagnosis, University of Pennsylvania; Professor of Pathology, Woman's Medical College of Philadelphia. Fourth edition, revised. Octavo of 670 pages. Philadelphia and London: W. B. Saunders & Company, 1905. Cloth $3.50 net.

The new fourth edition of Dr. Steven's excellent work on practical therapeutics appears at a most opportune time, close upon the issuance of the Eighth Decennial Revision of the Pharmacopœia to which it has been adapted. Dr. Stevens, by his extensive teaching experience, has acquired a clear, concise diction that adds greatly to his work's pre-eminence. New articles have been added on Scopolamin, Ethyl Chlorid, Theocin, Veronal, and Radium, besides much new matter to the section on Radiotherapy. The numerous changes in name or strength of various drugs and preparations, as called for by the new Pharmacopeia, have also been made. In fact, it is somewhat difficult to speak of Dr. Stevens' Therapeutics without resorting to the frequent use of superlatives, for of all the good works on this most important of subjects, this book before us is undoubtedly the very best.

